# Publisher Correction: Epidemiological characterization of SARS-CoV-2 variants in children over the four COVID-19 waves and correlation with clinical presentation

**DOI:** 10.1038/s41598-022-17020-6

**Published:** 2022-07-27

**Authors:** Claudia Alteri, Rossana Scutari, Valentino Costabile, Luna Colagrossi, Katia Yu La Rosa, Emanuele Agolini, Valentina Lanari, Sara Chiurchiù, Lorenza Romani, Anna Hermine Markowich, Paola Bernaschi, Cristina Russo, Antonio Novelli, Stefania Bernardi, Andrea Campana, Alberto Villani, Carlo Federico Perno

**Affiliations:** 1 Multimodal Research Area, Bambino Gesù Children Hospital IRCCS, Rome, Italy; 2 Department of Oncology and Hemato‑Oncology, University of Milan, Milan, Italy; 3 Microbiology and Diagnostics of Immunology Unit, Bambino Gesù Children Hospital IRCCS, Rome, Italy; 4 Academic Department of Pediatrics, Bambino Gesù Children’s Hospital, IRCCS, Rome, Italy; 5 Laboratory of Medical Genetics, Translational Cytogenomics Research Unit, Bambino Gesù Children Hospital IRCCS, Rome, Italy

Correction to: *Scientific Reports* 10.1038/s41598-022-14426-0, published online 17 June 2022

In the original version of this Article, reference 9 was erroneously cited twice. As a result, the subsequent references were incorrectly numbered. The reference list was correct at the time of publication.

The original Article has been corrected.

